# Post-Graduate Study

**Published:** 1892-04

**Authors:** R. B. Tuller

**Affiliations:** Chicago, Ill.


ARTICLE II.

### POST-GRADUATE STUDY.

BY R. B. TULLER, D. D. S., CHICAGO, ILL.

Read before the Chicago Dental Society.

The subject I have chosen to bring before you this evening, Post-Graduate Study, permits of wide range of thought, but the province of this paper is to present to you as briefly as I can some essential features that appear to me to be worthy of your serious consideration.

As it is said there is nothing new under the sun, I cannot hope to advance many ideas that are new, but shall content myself with the fact that in turning over some old ones, you will find the subject one that has not been so often revamped as some others in dentistry.

"Education ends only with life." Hence the most extensive period of our pursuit of knowledge comes after school days are over, and is therefore post-graduate study.

It would seem of importance then for us as dentists to thoroughly comprehend the situation, and in our aim to elevate the profession collectively and individually, to look at the facts as we find them.

We claim for dentistry the *rank* of profession, but it is a fact to be regretted that we are largely made up of men who never looked inside of a college, and that the average graduate in dentistry does not fulfill the conditions as to liberal education that one would expect to find in a professional man, or even a thorough and competent dentist in many cases.

As the same thing applies to graduates in other professions in this country, we are not alone in that.

We may all know why this is so and the remedy for it, but it is not my purpose to enter into the why and the wherefore. I believe the recognized colleges of dentistry have kept apace with the advancement of dentistry at large and will continue to do so in future. Everything of this character is one of the educational problems and has to grow and develop; it cannot spring to perfection at a bound.

I simply desire to show by the existing state of things what need there is to stimulate post-graduate study or *home* study to greater activity.

If existing methods have failed to advance students sufficiently, a way must be opened for further advancement by some other method, which will be adapted to such conditions as they find after leaving school, i. e., in practice.

Home study must be prosecuted during the leisure hours which we may have. To one in fair practice and attending to the many other duties of life, such leisure is not abundant. Whatever time we may have for study or reading, it is essential to know how to employ to the best advantage. One hour a day systematically employed would add much to one's store of knowledge in a year.

One must per force add something in daily practice whether especially sought after or not. We must do one thing or the other, advance or retrograde. We cannot stand still. The mind that is not developing is deteriorating. Experience is a good teacher, but life is too short to fill our measure in that way. To be successful we must keep up with the procession. To be broadly intelligent we must profit by the experience of those who have gone before, and who have woven the threads of knowledge which they found by long, diligent search and weary groping, into valuable literary fabric that others might be benefitted thereby. But we do not want to read at random. To be sure many men follow the bent of their own inclinations in educating themselves and improving their minds, ultimately reaching the higher rounds of the ladder. But that does not argue that they might not have had much more rapid progress and greater success under a properly directed and systematic course. We are not all alike and have not all the same capacity or power of comprehension and perception. Most of us need guidance and advice when we undertake to navigate unknown waters or follow unfamiliar paths. We are otherwise likely to drift.

The Chicago Dental Sociely has upon its rolls the names of many members who rank among the leaders in the dental profession and who have well earned that distinction.

I do not presume to come before such men and advise a course by which *they* may be better able to uphold the dignity of the profession; but we know that taking the profession at large, as it stands to-day, both as regards graduates and non-graduates, there is much to be desired in the way of advancement all along the line, and I am imbued with the idea that a regular, systematic course of post-graduate study or reading can and should be established, arranged for and open to all kinds and conditions of men now in actual and legitimate practice of dentistry, and that it would do more eventually to elevate the profession at large than all the laws or all the influences that have so far been brought to bear, except, perhaps, that of good dental periodicals, and we should hope to have their valuable assistance in this work.

Dentistry being largely a development of the last half a century, and dental colleges a sequence in the progress, and there being a necessity for restrictive laws not only enacted but enforced—not yet fully comprehended it seems: it is not surprising to find our profession largely made up of practitioners without diplomas. I think some may be surprised to know that out of about eighteen or twenty thousand dentists now in practice in the United States, less than one-third are graduates. The data for this statement comes from reliable gentlemen in the profession who have taken pains to get at the truth.

If what an eminent member of our profession, an M.D.,

D. D. S., in correspondence with me, says is true, a diploma does not always indicate the better man or dentist. I will quote from his letter: "The average student is after a diploma and nothing more. When he has acquired it he at once proceeds diligently to forget all that he has learned, rather than to acquire any new knowledge. He don't want scholarship, he merely wishes the *name* of it."

I believe that is true of many. The hustle after the nimble dollar absorbs all other ideas. To a greater or lesser extent it cuts a figure in the affairs of all men.

But many men become indifferent to advancement, because circumstances have cut them off from environments that are conducive to the ever onward and upward. We in large cities where it is easy to convene from time to time for mutual improvement are more fortunate. In such intercourse there is that healthy competition of active minds that stimulates all to greater exertion and higher aims. The value of such personal contact cannot be overestimated.

Let me quote a little from Henry Drummond's "Natural Law": "The development of any organism in any direction is dependent on its environments. A living cell cut off from air will die. A seed germ apart from moisture and an appropriate temperature will make the ground its grave for centuries. Human nature, likewise, is subject to similar conditions. It can only develop in presence of its environments. No matter what its possibilities may be, no matter what seed of thought and virtue, what germs of genius or of art lie latent in its breast, until the appropriate environment presents itself the correspondence is denied and development discouraged, the most splendid possibilities of life remain unrealized, and thought and virtue, genius and art are dead."

The "Chautauqua idea," which I believe originated the plan of systematic home study, is not new in connection with dentistry. Dr. W. C. Barrett, of Buffalo, N. Y., urged a movement of this kind as long ago as 1884, and shortly after made a very urgent plea for it before a dental convention at Springfield, Mass. In the June number of the *Independent Practitioner* for 1886 he said: "Is it not practicable to establish a grade of study to be pursued at home, to mark out a systematic course to be studied under competent instructors, the tuition to be obtained by correspondence or by the publication of lectures on definite themes somewhat after the manner of the Chautauqua literary course, but modified to suit the exigencies demanded."

There are difficulties in the way no doubt. There are difficulties in the way of almost any undertaking. If the idea is good that should not deter us. Under proper auspices, seems to me such a course ought to become popular with every dentist in the profession. It should have the good-will and co-operation of colleges and college graduates, for in no way would it conflict with their work, and in no sense could it be construed as a substitute for college training.

There can be no injustice to graduates that I can see in opening our course to non-graduates. Many non-graduates are in every way worthy men and many of them practiced dentistry before a good many of the colleges came into existence, and when we think of their large number let us draw no line except to extend no benefits to any not now in the profession.

Now, in establishing a post-graduate course of study, some new degree, or something to signify reward of merit, must be adopted. It must not be a cheap distinction, but must represent real culture and attainment which may be acquired by a faithful study of the course.

I have corresponded with quite a number of prominent men in the profession, and so far have not found one who does not speak favorably of such a movement if it can be properly directed.

One writes me at considerable length and I will read a portion of what he says. It perfectly accords with my own views, and is so much better expressed than I could do. He says:

"In the first place I should expect but a limited number to begin with this course. We have too few real students to get many earnest men. But I would have a convention of some kind, the most experienced educators in dentistry being in attendance, and after due deliberation a schedule of real study extending over some years—three at least—adopted, and text books recommended. I would have a competent corps of teachers appointed, under whose advice and instruction every student should study.

Of course this must be done by correspondence. * *

Every year there should be a finishing course of lectures delivered upon definite subjects within the studies of the year, and a careful examination held by the teachers, or better still by a competent board, if such an one could be organized.

Let the examinations be *rigid* and *exhaustive* and incompetent men be mercilessly plucked. Then there would be some credit in passing the examination, and the thing would not be a farce.

This annual meeting could take the place of a dental society meeting. It might last a week or more. The very best men in the several departments, the most experienced teachers could be engaged. They could get some fees, for it is idle to believe that the *best* men could be expected to give their time free. Fees could be charged for passing and finishing.

If some plan like this could be rigidly enforced and the examinations made something more than a mere farce, the annual meetings would become one of the greatest events of the year. It would be a gathering of the *scholars* of the profession, and very soon all the scholars would be found there. The lectures and demonstrations (not clinics) by the very best men outside or inside the profession would attract the attention of all and would insure the regard of the scientific world.

In time such an organization, if conservatively managed, would occupy a position in dentistry analagous to that which the Royal Society of England, and the Academy Francaise of France hold, and membership would be an appreciated honor. But this could only be accomplished by a very exclusive course. Anything which should be done simply for the purpose of securing popularity would be ruinous to the final end."

This seems to point out how a post-graduate degree might be adopted and made one of high spirit.

In my own way of thinking the course should embrace everything from the fundamentals of dentistry up to anything that might be demanded at the top to make men broadly intelligent outside of dentistry, and should be divided into series or classes, with certificates granted at the completion of each series. Those who need to go through the entire course could do so, but graduates in dentistry and those further advanced might begin according to their advancement.

I wish, gentlemen, this subject might have been presented to you by some one better fitted for it than I am, for I am one of those who most thoroughly feel the need of post-graduate study and do not pretend to be a leader in such a movement. While it has interested me greatly and I shall do what I can in my humble way to further the cause, I am seeking for some one who is well qualified to step forward as its champion. With so much need of it and so much to commend it, I hope to see a post-graduate course established. But a handful of somewhat obscure men cannot, I fear, accomplish it. It must have the co-operation and indorsement of the best men in the profession and the course of study must of course be directed by the most competent men.

The Post-Graduate Dental Association of the United States was organized with this movement as one of its aims and objects. They are so unfortunate, as many of you know, as to have your humble servant as the present

President. About the only quality I pretend to possess concerning this matter is persistence in trying to bring this movement to the front, and I am thus open to your criticisms, and I shall feel that something has been gained if I can get any suggestions or new ideas that will help along the cause.—*Dental Review.*

